# AI-enhanced genomic interpretation in Wilson’s disease: a step toward earlier detection

**DOI:** 10.1097/MS9.0000000000004409

**Published:** 2025-11-21

**Authors:** Hasibullah Aminpoor, Muhammad Khizar, Muhammad Zaib, Hasiba Karimi, Muzamil Khan

**Affiliations:** a Faculty of Medicine, Kabul University of Medical Sciences “Abu Ali Ibn Sina”, Kabul, Afghanistan; b Faculty of Medicine, Georgian American University, Tbilisi, Georgia; c Manifest Medical Research Co, Tbilisi, Georgia; d Faculty of Medicine, Bezmialem Vakif University, Istanbul, Turkey; e Internal Medicine, The George Washington University School of Medicine and Health Sciences, Washington, DC, USA

*To the Editor,*

Wilson’s disease (WD) is a rare inherited disorder caused by mutations in the ATP7B gene, leading to copper accumulation in the liver, brain, and other organs^[1]^. If untreated, copper accumulation can result in liver cirrhosis, acute liver failure, or neurological and psychiatric problems^[1]^. This diagnostic challenge is particularly relevant in populations with high rates of consanguinity, such as in India^[2]^.

Current diagnosis relies on clinical features, including Kayser–Fleischer rings, laboratory tests like low serum ceruloplasmin and elevated urinary or hepatic copper, and, increasingly, genetic testing following guidelines such as the Leipzig criteria^[3]^. Limitations persist: vague early symptoms may lead to misdiagnosis^[4]^, and liver biopsy, though the gold standard, can yield false negatives if performed early and carries procedural risks^[5]^

The genetics of WD is complex, with over 900 reported ATP7B variants and many variants of uncertain significance (VUS)^[2]^. Manually classifying these variants is time-consuming, which is where artificial intelligence (AI) can add value. For instance, Vatsyayan *et al* (2024) developed “WilsonGenAI,” a deep learning tool trained on a large variant database, achieving nearly 99% accuracy in identifying known pathogenic variants and reclassifying numerous VUS^[1]^. More generally, AI can find patterns in complex datasets that are difficult for humans to recognize, which is especially helpful for rare diseases^[6]^. AI models that integrate genetic, laboratory, and clinical data show promise for detecting WD before overt symptoms appear^[3]^

Our perspective is that beyond automating variant classification, AI can serve as a proactive clinical tool: by combining genomic data with electronic health records, it could flag at-risk individuals for early testing and intervention. This approach could also inform population-level strategies in regions with higher disease prevalence.

However, several limitations must be acknowledged. AI models are susceptible to data bias, overfitting, and reduced generalizability, particularly in rare diseases where datasets are small^[6]^. Multicenter validation and careful model calibration are therefore essential before clinical implementation.

In conclusion, AI-enhanced genomic interpretation represents a promising approach for earlier detection of WD. Collaboration between clinicians, geneticists, and data scientists will be important to refine these tools and incorporate them into practice. Early identification of WD through AI has the potential to improve patient outcomes significantly. By integrating multiple data types and supporting proactive clinical decision-making, it has the potential to overcome current diagnostic challenges. Collaborative efforts between clinicians, geneticists, and data scientists will be crucial to refine these tools and ensure safe, effective translation into practice.

Our work is in line with the TITAN Guidelines on the need for transparency in AI use in healthcare^[7]^.

## Data Availability

Not applicable.
